# Characterizing the BCG Induced Macrophage and Neutrophil Mechanisms for Defense Against *Mycobacterium tuberculosis*

**DOI:** 10.3389/fimmu.2020.01202

**Published:** 2020-06-18

**Authors:** Thomas E. Bickett, Jennifer McLean, Elizabeth Creissen, Linda Izzo, Cassidy Hagan, Antonio J. Izzo, Fabiola Silva Angulo, Angelo A. Izzo

**Affiliations:** Department of Microbiology Immunology and Pathology, Cell and Molecular Biology, Colorado State University, Fort Collins, CO, United States

**Keywords:** innate, BCG—Bacille Calmette-Guérin vaccine, macropahge, mycobacerium tuberculosis, lung

## Abstract

The live attenuated *Mycobacterium bovis* strain, Bacille Calmette Guérin (BCG) is a potent innate immune stimulator. In the C57BL/6 mouse model of tuberculosis, BCG vaccination leads to a significant reduction of *Mycobacterium tuberculosis* burden after aerogenic infection. Our studies indicated that BCG induced protection against pulmonary tuberculosis was independent of T cells and present as early as 7 days after vaccination. This protection showed longevity, as it did not wane when conventional T cell and TNF-α deficient mice were infected 30 days post-vaccination. As BCG induced mycobacterial killing after 7 days, this study investigated the contributions of the innate immune system after BCG vaccination to better understand mechanisms required for mycobacterial killing. Subcutaneous BCG inoculation resulted in significant CD11b^+^F4/80^+^ monocyte subset recruitment into the lungs within 7 days. Further studies revealed that killing of mycobacteria was dependent on the viability of BCG, because irradiated BCG did not have the same effect. Although others have identified BCG as a facilitator of trained innate immunity, we found that BCG reduced the mycobacterial burden in the absence of mechanisms required for trained innate immunity, highlighting a role for macrophages and neutrophils for vaccine induced killing of *M. tuberculosis*.

## Key Points

BCG induces potent innate immunity against M. tuberculosis pulmonary infection, Immunity is dependent on neutrophil and CD11b^+^F4/80^+^ monocyte recruitment, Viable BCG is required for killing of *M. tuberculosis*.

## Introduction

Bacille Calmette Guérin (BCG), is a potent immune response modifier used as a vaccine against *Mycobacterium tuberculosis* infection, a treatment for bladder cancer (1), as well as an adjuvanted delivery platform for vaccines against other pathogens (2). Since these findings, the concept of “trained-immunity” as a means of explaining the phenomenon has been proffered and shown that BCG induces epigenetic changes in human monocytes (3–5) as well as reside in bone-marrow derived cells (6). These data provide strong evidence to suggest that BCG is a potent biological response modifier but leave many questions unanswered. It has been our experience that in the C57BL/6 model of experimental tuberculosis, BCG vaccination resulted in a significant reduction in mycobacterial growth when mice were infected with virulent *M. tuberculosis*, 30 days after vaccination. Induction of adaptive immunity has been demonstrated by us and others and is independent of the sub-strain of BCG (7). However, BCG induced adaptive immunity is limited and did not correlate to the reduction in mycobacterial growth (8). We hypothesized that a significant contribution to *M. tuberculosis* killing after BCG vaccination was provided by early recruitment of macrophages as a part of the innate immune mechanisms, and that this established an environment to induce adaptive immunity that was responsible for prevention of disease.

BCG is known to stimulate multiple pathogen recognition receptors (PRRs) such as Toll-Like Receptors (TLR)-2/4/9 and nucleotide-binding oligomerization domain-like receptors (NOD) (3, 9), suggesting that BCG provides multiple potent signaling mechanisms to innate myeloid and lymphoid cells. Cells such as dendritic cells (DCs), monocytes, macrophages, neutrophils and innate lymphoid cells (ILCs) may be triggered by BCG to be activated and subsequently induce T cell mediated immunity that is required to kill *M. tuberculosis*. We asked, what if these cells had the capacity to kill *M. tuberculosis* prior to the induction of adaptive immunity, which of these cells was responsible, and how were they performing their function? Recent studies demonstrated that BCG can cause epigenetic changes in macrophages, and BCG infected bone marrow-derived macrophages, when adoptively transferred, reduced the mycobacterial burden in infected recipients (6). However, these studies did not address which cells were directly responsible for killing *M. tuberculosis* and what mechanisms were used for killing. In the current studies, the mouse model was redesigned from the standard 30 day post-vaccination model (10) to examine the immune response within 7 days of vaccination, prior to induction of adaptive immunity, to determine how innate immune mechanisms affect the growth of *M. tuberculosis*. Using a series of depletion and knockout (KO) mouse studies, we demonstrate that after subcutaneous BCG inoculation, neutrophils, circulating monocytes, and alveolar macrophages are sufficient to reduce mycobacterial burden, and that live BCG was required to stimulate this immune response.

The PRR NOD2, is of significant importance to BCG mediated immunity as recent evidence has suggested that cells of the innate immune system can exhibit a memory like response through recognition of BCG with the NOD2 receptor (3–5). Upon stimulation with muramyl dipeptide (MDP), the NOD2 receptor induces epigenetic reprogramming of the genes responsible for initiating inflammation so that a second stimulation with MDP can produce a more robust and quicker response leading to better clearing of the pathogen in what has been called trained innate immunity (3–5, 11). Studies in humans deficient for NOD2 have shown that macrophages were unable to properly activate the inflammasome for IL-1β signaling (12, 13) and showed reduced capacity to produce TNF-α (3), indicating a potential for this phenomenon to translate to humans. If so, the BCG vaccine may have certain benefits for human use and requires a better understanding of the mechanisms by which it can induce mycobacterial killing in the mouse model.

Studies have demonstrated that BCG can induce strong T cell responses (14, 15), but our work and others have suggested that it is unclear whether or not this directly correlates to protection against *M tuberculosis* (8). Many new vaccines for *M. tuberculosis* are currently in clinical trials, and a number of them still use BCG in some form. The BCG vaccine has a future in tuberculosis prevention, but without a full understanding of the mechanism of action behind its induction of protective immunity it may not be utilized properly. Recent publications have highlighted the importance of the innate immune system during BCG vaccination, and have indicated that non-specific effects of BCG vaccination may benefit young children even if protection against *M. tuberculosis* is not attained (3, 16–18). In 2012 Aeras and the TuBerculosis Vaccine Initiative published a strategic blueprint for the next decade of tuberculosis vaccines, which called for research into alternative mechanisms for dealing with infection of *M. tuberculosis* (19). It is interesting that BCG has been in use for so long without an adequate understanding of the types of immune responses it generates, and as the current study demonstrates there is much yet to be learned. Given that BCG is the most widely used vaccine in the world, administered to millions of infants annually, it is imperative to understand the effects that BCG has on the innate immune system while remaining viable and possibly proliferating. Although others have explored how BCG interacts with other innate immune cells, the majority have focused on adaptive immunity (20). This study focuses on the unconventional, T-cell independent mechanisms induced by the BCG vaccine.

## Materials and Methods

### Mice

Female C57BL/6, B6.129S2-Cd4^*tm*1*Mak*^/J (CD4KO), B6.129S2-Cd8a^*tm*1*Mak*^/J (CD8KO), B6.129S-Tnf^*tm*1*Gkl*^/J (TNF-α KO), and B6.129P2-Lyz2^*tm*1(*cre*)*Ifo*^/J mice (LyzMcre KO) (6–8 week-old) were purchased from Jackson Laboratories (Bar Harbor, ME) and maintained at CSU for 2 weeks prior to experimentation. Nod1^−/−^/Nod2^−/−^ (NOD1/NOD2- deficient) mice were a kind gift from Dr. Andreas Baumler (University of California, Davis), and were described previously (21). The Institutional Animal Care and Use Committee at Colorado State University reviewed and approved all experiments (Protocol ID: 16-6369A).

### Mycobacteria

*M. bovis* BCG Pasteur (TMC#1011) was grown in Proskauer and Beck (P&B) medium containing 0.01% Tween® 80 until mid-log growth phase. It was then aliquoted and stored at −80°C for future use. *M. tuberculosis* H37Rv (TMC# 102) was grown in P&B medium as a pellicle and then transferred to liquid cultures of P&B medium containing 0.5% Tween® 80. The cultures were passaged three times through the same media grown in a roller bottle incubator and finally aliquoted and stored at −80°C. The number of colony forming units (CFU) and viability of all cultures was assessed after freezing and was >95% viable. The genome of the BCG Pasteur used in these studies was sequenced (Dr. M. Strong, Center for Genes, Environment and Health, National Jewish Health and University of Colorado, Denver, CO) and was >99.99% similar to the published reference genome for BCG Pasteur (22).

### BCG Vaccination

BCG was diluted to a concentration of 5 × 10^5^ CFU/mL with sonicating to disrupt clumps. The vaccine was loaded into 1 mL syringe and 100 μL was inoculated to deliver 5 × 10^4^ CFU subcutaneously, 1 × 10^6^ CFU intravenously, 1 × 10^7^ intranasal, 5 × 10^4^ intramuscular per mouse. Low dose aerosol BCG vaccine given at 50–100 CFU per mouse. The inoculum was plated on 7H11 agar (Difco, BD Biosciences, San Jose, CA) to assess the accuracy of vaccine dose. Mice were rested for 7 days post-vaccination and then infected with *M. tuberculosis*. In some studies mice received BCG that was exposed to 2.4mRads of γ-irradiation from Cs^137^ source (Irradiation Services Laboratory, CSU) that kills the organism, as determined by plating after irradiation procedure (data not shown) and has been demonstrated by others to be metabolically active (23).

### *M. tuberculosis* Infection

*M. tuberculosis* H37Rv was diluted to a concentration of 2 × 10^6^ CFU/ml with prior sonication to separate aggregates. Five milliliter was then loaded into a nebulizer in order to deliver 50–100 CFU to each mouse using the Inhalation Exposure System (Glas-Col, Terre Haute, IN) according to the established laboratory protocols. The inoculum was plated on 7H11 agar in addition to 5 mice being sacrificed on day of infection to assess the number of CFU implanted into the lung.

### Assessment of Bacterial Burden

CFU in the lungs and spleens of mice were determined at 30 days post-infection. Necropsy was performed to remove organs which were then homogenized in sterile saline before being diluted and plated on 7H11 agar. The plates were incubated at 37°C for 14–18 days. Colony counts were converted into Log_10_ CFU for analysis. All CFU were performed with five mice per group unless specified otherwise.

### Antibody Depletion

Mice were depleted of neutrophils through intraperitoneal injection of 200 μg anti-Ly6G antibody (clone 1A8; Bio X Cell, Lebanon, NH) in PBS every 4 days. Mice were depleted of Natural Killer (NK) cells through intraperitoneal injection of 250 μg anti-asialo GM1 antibody (Accurate Chemical, Westbury, NY) in PBS every 3 days. In one set of experiments, mice were vaccinated subcutaneously 3 days after the first depletion of neutrophils so that neutrophils would not be present during vaccination with 5.0 × 10^4^ CFU BCG Pasteur. Thirty days post vaccination, mice were infected with a low dose aerosol of *M. tuberculosis*, H37Rv as described. In a second set of experiments, mice were depleted of NK cells starting 3 days prior to infection so that NK cells would not be able to contribute to mycobacterial killing during infection. Control mouse groups received the appropriate isotype antibody at the same concentration. Cell depletion efficiency was assessed by flow cytometry using anti-NK1.1 for NK cells and anti-Ly6G for neutrophils (Supplementary Table 1).

### Flow Cytometry

Single cell suspensions were prepared from lungs that were minced with a razor blade before being incubated at 37°C in 0.5% Liberase (Sigma Aldrich, St. Louis, MO) incomplete RPMI solution (Life Technologies, Carlsbad, CA). After 45 min lung pieces were passed through a 70-μm nylon cell strainer (Falcon, Corning, Durham, NC) and the single cell suspension collected by centrifugation. The pelleted cells were resuspended in 2 mL ACK red blood cell lysis buffer (Life Technologies, Carlsbad, CA) and incubated at room temperature for 5 min before 10 mL of complete RPMI (RPMI-1640 with essential and non-essential amino acid, penicillin, streptomycin, HEPES (Sigma Aldrich, St. Louis, MO), sodium pyruvate (Sigma Aldrich, St. Louis, MO), L-glutamate (Sigma Aldrich, St. Louis, MO), and 10% fetal bovine serum (FBS) (Atlas Biologicals, Fort Collins, CO) was added to stop the reaction. Cells were centrifuged and stored in complete-RPMI on ice while cells were counted and the concentration adjusted to 2 × 10^6^/mL in complete-RPMI. Cells were pelleted and incubated for 20 min at 4°C in 2.4G2 hybridoma supernatant (Fcγ blocking antibody; ATCC® HB-197) diluted in PBS containing 5% FBS 0.01% NaN_3_ (FACS buffer). Cells were washed by centrifugation and resuspended in clean FACS buffer before being pelleted and stained with the fluorochrome-conjugated antibodies (Supplementary Table 1). Cells were washed again with FACS buffer before being analyzed on a BD FACS Canto II flow cytometer and data analyzed using FlowJo software (FlowJo, LLC, Ashland OR).

### Macrophage Stimulation Assay

RAW Blue^TM^ macrophage reporter cells (InvivoGen, San Diego, CA) engineered with a chromosomal integration of a secreted embryonic phosphatase reported construct inducible by NF-κB and AP-1 were used to assess the activity of BCG on macrophages. Cells were cultured in complete RPMI (Life Technologies, Carlsbad, CA) in 24 well tissue culture plates (Corning Incorporated, Corning, NY) and stimulated with either live BCG Pasteur or γ-irradiated BCG Pasteur. Supernatants were taken and the amount of alkaline phosphatase was measured by addition of QUANTI-blue^TM^ (InvivoGen). Supernatant was then transferred to a 96 well plate and optical density was measured at 72 h at 630 nm.

### Preparation of Bone Marrow Derived Macrophages (BMDM) and Real-Time PCR Analysis

Bone marrow cells were harvested from C57BL/6 mice and added to complete RPMI containing 20 ng/mL M-CSF (macrophage colony-stimulating factor; Peprotech, Rocky Hill, NJ) to drive macrophage differentiation. Media was changed every 72 h until the eighth day in which media was changed to exclude M-CSF and antibiotics. On day 7, cells were harvested and brought to a concentration of 1.0 × 10^6^ macrophages per well in a 24 well plate for stimulation for 24 and 48 h with varying MOI of BCG or irrBCG. Supernatants and total RNA was harvested at each time point. RNA was isolated using TRIZol® (Invitrogen), and quantified using a Nanodrop Microvolume Spectrophotometer (Thermo Fisher Scientific). RNA was then converted into cDNA using an iScript cDNA synthesis kit (BioRad, Hercules, CA), and RT-PCR was performed using the Qiagen RT^2^ Profiler^TM^ PCR Array for Mouse Cytokines and Chemokines on a CFX Connect^TM^ Real-Time PCR Detection System (BioRad). Data were analyzed using the Qiagen online Data Analysis Center. The data generated from the PCR array is available at https://www.ncbi.nlm.nih.gov/geo/query/acc.cgi?acc=GSE139864.

### Cytokine Analysis

Cytokine quantification in cell culture supernatants following stimulation was performed by enzyme-linked immunosorbent assay (ELISA). ELISA kits (Affymetrix/eBioscience INC San Diego, CA) for the following cytokines: TNF-α, IL-1β, IL-6, and IL-10 were used following the manufacturer's protocol. The color intensity in wells of 96 well plates was then read using the Ultramark^TM^ Microplate Reader (BioRad, Hercules, CA). A standard curve was also used with each assay to determine cytokine concentration in pg/mL. Lung homogenates from infected mice were pelleted and the cell free supernatant was taken to quantify cytokine concentrations, and a Cytometric Bead Array (CBA) assay was performed using CBA mouse inflammation kit (BD Biosciences, San Jose, CA) on lung supernatants. Data from the bead assay were collected on a FACSCanto II cytometer (BD Biosciences, San Jose, CA) according to protocol with kit and analyzed using FCAP Array^TM^ software (BD Biosciences).

### Statistical Analysis

Data were analyzed using the statistical tests as described using R software (R foundation). Data from some experiments were Log_10_-transformed prior to analysis. Graphs were prepared using Graph Pad Prism 7 (GraphPad software, La Jolla, CA).

## Results

### CD4/CD8/TNF-α Independent *M. tuberculosis* Killing Mechanisms Induced by BCG

**Figure 1:**
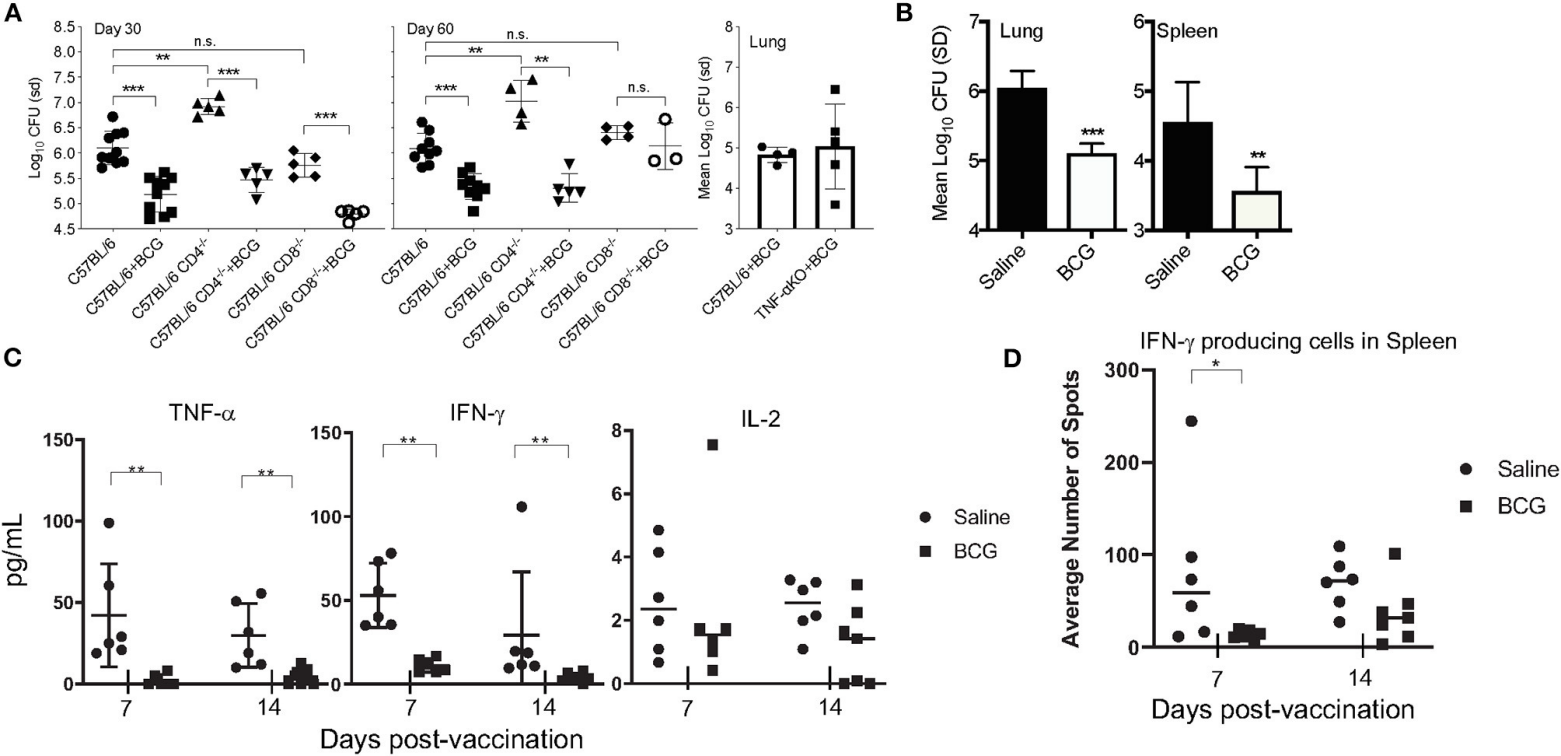
C57BL/6 CD4, CD8, and TNF-α knockout mice were vaccinated subcutaneously with 5 × 10^4^ CFU BCG, rested for 30 days and then infected with a low dose aerosol of *M. tuberculosis* H37Rv. CFU were determined at days 30 and 60 post-infection for the CD4^−/−^ and CD8^−/−^ mice and day 30 for the TNF-α KO mice **(A)**. C57BL/6 mice vaccinated with 5 × 10^4^CFU BCG Pasteur 7 days before infection and the CFU determined 30 days after infection in lungs and spleens **(B)**. The concentration of and TNF-α, IFN-γ, and IL-2 as determined by cytometric bead array (CBA) at day 30 post-infection when mice were vaccinated 7 and 14 days infection **(C)**. IFNγ producing cells present in spleen as determined by ELISpot after stimulation with H37Rv Culture filtrate protein (CFP) after infection when mice are vaccinated 7 and 14 days before infection **(D)**. Experiments were performed with *N* = 4–5 mice per group and are representative of multiple iterations. *p ≤ 0.05 **p ≤ 0.01, ***p ≤ 0.001, n.s., not significant.

In the mouse model, BCG is well-established at conferring protective immunity against experimental pulmonary *M. tuberculosis* infection. To determine the role of innate immunity induced by BCG, CD4KO and CD8KO mice were vaccinated with 5 × 10^4^ CFU of BCG Pasteur 30 days prior to infection, the time at which adaptive immunity is active. Mice vaccinated with BCG 30 days prior to infection also showed a 1 Log_10_ reduction in CFU in lungs (Figure 1A) at days 30 and 60 post-infection, representing a 90% reduction in mycobacterial burden in the absence of either conventional CD4^+^ or CD8^+^ T cell mediated immunity. We then wanted to determine if TNF-α, a key cytokine required for protective immunity was essential for killing *M. tuberculosis* after BCG vaccination. Without BCG vaccination, mice have been shown by others to succumb to infection within 30 days of a low dose aerosol infection with *M. tuberculosis* H37Rv (24). No significant difference was observed between BCG-vaccinated C57BL/6 and TNF-α KO mice at day 30 post-vaccination (Figure 1A). Our data showed that three major adaptive immune elements were not an absolute requirement for BCG mediated killing of *M. tuberculosis*.

### BCG-Induced Innate Immune Mechanisms Are Sufficient to Kill *M. tuberculosis*

We then altered the paradigm to ask if a similar reduction in CFU was observed at an earlier time (prior to induction of T cell mediated immunity) in immunocompetent mice. To this end, when mice were vaccinated 7 days before pulmonary infection, there was a significant reduction in CFU in the lungs and spleens of mice (Figure 1B). We chose to examine lung CFU at day 30 post-infection because it is our experience that it is difficult to observe a significant difference between BCG vaccinated and naïve mice prior to day 21 of infection, even at day 30 post-vaccination, when adaptive immunity has been stimulated by vaccination (Supplementary Figure 3). As 7 days was not a sufficient time to mount an adaptive immune response, this led us to hypothesize that innate immune cells significantly contributed to the BCG induced reduction of the mycobacterial burden. To better understand the nature of the early immune response in the lungs, cytokine analysis was performed on lung tissue at day 30 of infection (Figure 1C). IFN-γ and TNF-α were significantly reduced in the lungs of mice that had been vaccinated at either 7 or 14 days prior, compared to non-vaccinated mice that were subjected to the same infection, suggesting a non-essential role for these cytokines in BCG induced immunity. T cell activation at days 7 and 14 post-vaccination, examined by ELISpot assay revealed no difference in antigen-specific T cells in the spleens between vaccinated and non-vaccinated, naïve mice (Figure 1D).

### BCG-Induced Macrophage Changes in the Lung After Subcutaneous Vaccination

**Figure 2:**
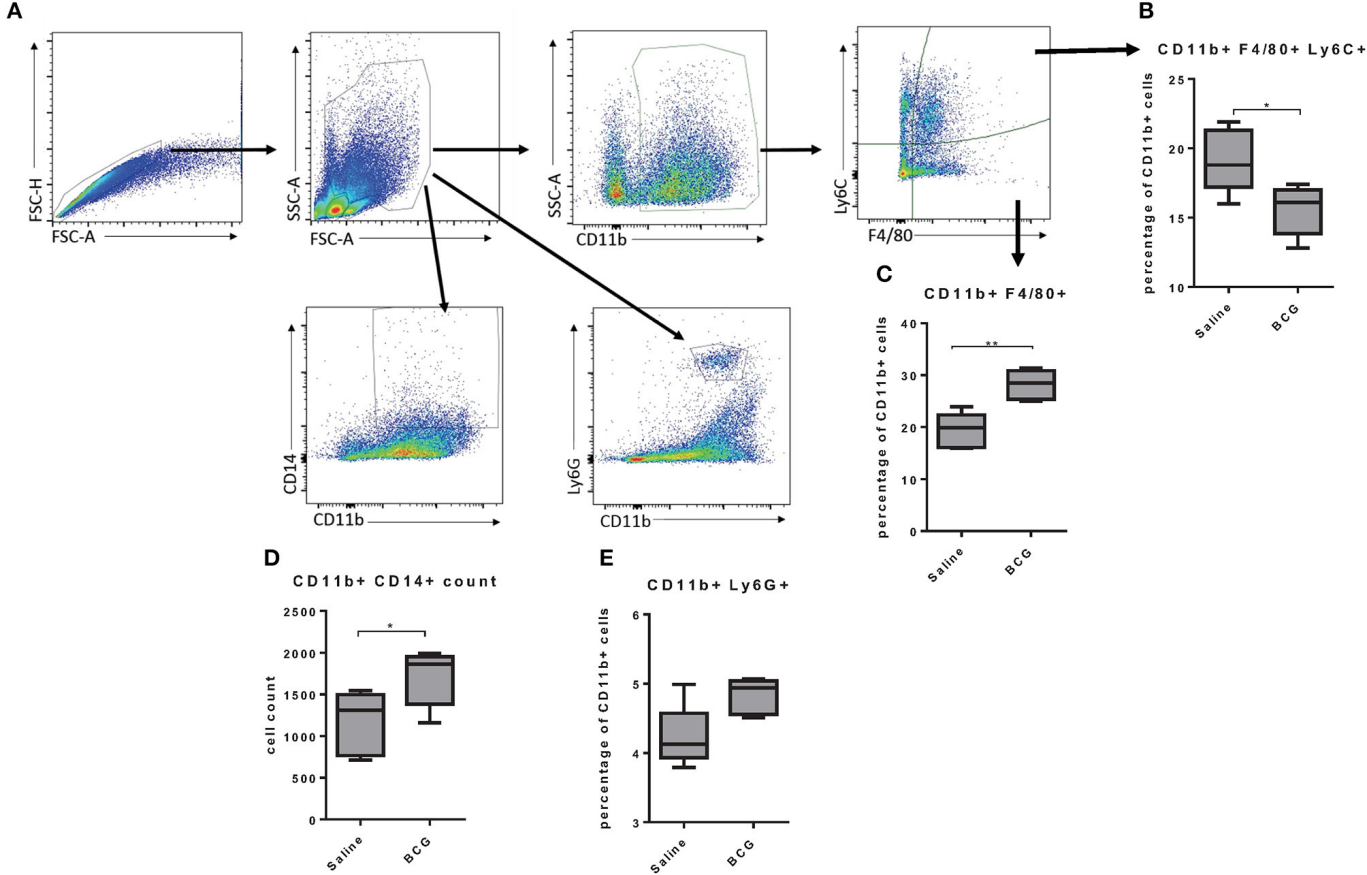
Flow cytometry gating strategy; singlets were identified using FSC-A and FSC-H, and granulocytes were gated based on size and granularity. The markers CD11b, Ly6C, and F4/80 were used to identify subpopulations of macrophages and Ly6G was used to identify neutrophils **(A)**. C57BL/6 mice, vaccinated subcutaneously with 5 × 10^4^ CFU BCG Pasteur, showed an increase of CD11b^+^ cells in the lungs and a lower percentage of those cells are positive for the marker F4/80 and Ly6C at day seven post-vaccination **(B)**. The lungs from BCG vaccinated mice also showed an increase in the percentage of CD11b^+^ cells that were also F4/80^+^ **(C)**. BCG vaccinated mice also showed an increase in the number of double positive cells in the lungs for CD11b and CD14 expression **(D)**. Lungs from vaccinated mice also had a small increase in CD11b^+^ Ly6G^+^ neutrophils **(E)**. *N* = 5 mice per group and are representative of multiple iterations, *p ≤ 0.05 **p ≤ 0.01 (Wilcoxon rank sum test).

As BCG induced protective immunity within 7 days we wanted to better understand the mechanism by which a subcutaneous vaccination induced protection in the lungs in that time. Others have reported that subcutaneous BCG vaccination induced systemic responses, such as increased CD14^+^ monocytes (3), but the response in the lungs has not been defined. We assessed changes in lung cell populations 7 days after subcutaneous BCG vaccination. Lungs from vaccinated and non-vaccinated mice were collected to identify differences in monocyte/macrophage populations using CD11b, Ly6C, F4/80, and CD14 markers (Figure 2A). Further analysis of the CD11b^+^ population showed that vaccinated mice had higher percentage of CD11b^+^F4/80^+^ cells but a lower percentage of CD11b^+^F4/80^+^Ly6C^+^ cells (Figures 2B,C). The Ly6C marker has been used to distinguish circulating monocytes vs. cells migrating into tissues (25) suggesting that upon BCG vaccination, the lungs experience an infection like state in which monocytes extravasate from the blood to help control the infection, although in this case in the absence of a lung infection. There is evidence that circulating monocytes can differentiate into interstitial macrophage populations once they reach tissue such as the lungs that have the capability to self-renew (25, 26), suggesting a possible mechanism by which BCG induces innate immunity in the lungs. There was also a significant increase in CD11b^+^CD14^+^ cells in the lungs of vaccinated mice (Figure 2D), that has been observed in the spleen by others (3). There was also a very minimal increase of neutrophils (CD11b^+^Ly6G^+^) in the lungs (Figure 2E). Our data suggested that BCG stimulation increased phagocytic cells in the lungs, but the role they played in protection against *M. tuberculosis* infection was unclear.

### Innate Immunity Against *M. tuberculosis* Infection, Induced by BCG Requires Neutrophils

**Figure 3:**
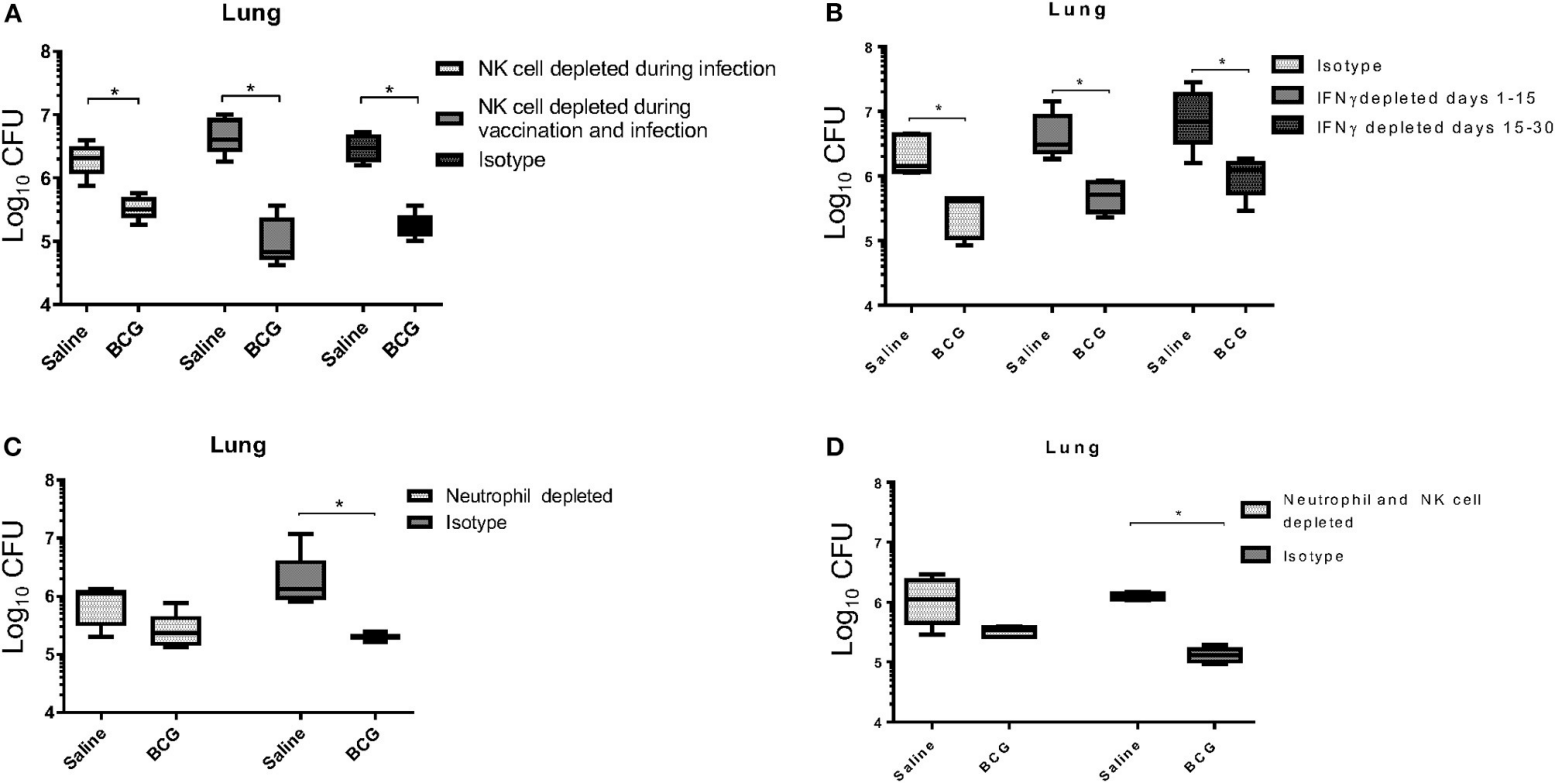
BCG vaccinated C57BL/6 mice, depleted of NK cells using anti-asialo GM1 antibody during infection and during the vaccination and infection periods, were able to significantly reduce the mycobacterial lung burden similar to isotype antibody-treated control mice **(A)**. NK cell depletion was confirmed through flow cytometry 3 days after depletion (Supplementary Figure 1B). C57BL/6 mice were vaccinated subcutaneously with 5 × 10^4^ CFU BCG Pasteur, and then depleted of IFN-γ during infection at either days 1 to 15, or 15 to 30 post-infection had significantly reduced mycobacterial lung burdens, similar to isotype antibody-treated infected control mice **(B)**. BCG vaccinated C57BL/6 mice, depleted of neutrophils during vaccination and up to 4 days post-vaccination, reduced their lung mycobacterial burden by ~0.5 Log_10_ CFU compared to isotype antibody-treated infected control mice **(C)**. Neutrophil depletion was confirmed through flow cytometry 3 days after depletion (Supplementary Figure 1A). BCG vaccinated C57BL/6 mice, depleted of neutrophils during vaccination, and up to 4 days post-vaccination, and NK cells from the time of infection, had reduced their mycobacterial lung burden by 0.5 Log_10_ CFU compared to isotype antibody-treated infected control mice, which are able to reduce burden by 1 Log_10_ CFU **(D)**. *N* = 5 mice per group for all experiments with two iterations, *p ≤ 0.05 (*T*–test).

It is generally accepted that IFN-γ produced by T cells is essential for controlling *M. tuberculosis* infection, through activation of infected cells such as macrophages (27, 28), although a prior study identified an IFN-γ independent mechanism for killing mycobacterium, that was however CD4 T cell-dependent (29). Our data support a previously published report that showed CD4^+^ T cells were not required for BCG induced mycobacterial reduction (30). As a T cell mediated IFN-γ response was not detected at this time to induce killing of mycobacterium (Figure 1D) our first approach was to target a potential source of IFN-γ in the innate immune system. In addition to their ability to produce IFN-γ, we were also interested in the role of Natural Killer (NK) cells during BCG vaccination for their contributions in controlling intracellular pathogens, especially *M. tuberculosis* (31). To better understand their role, we used anti-asialo-GM1 antibody to deplete NK cells in mice during infection with *M. tuberculosis* in both vaccinated and unvaccinated mice. In one scenario, mice were injected with anti-asialo-GM1 during the BCG vaccination, and infection periods, every 3 days until the day 30 time of necropsy. In a second scenario, mice were injected with anti-asialo-GM1 after *M. tuberculosis* infection only. Our data suggested that, regardless of when NK cells were depleted, they did not play a role in killing *M. tuberculosis* in either group (Figure 3A). Depletion of IFN-γ after aerosol infection, either immediately after or from day 15 post-infection, resulted in a significant reduction in the mycobacterial burden, suggesting the presence of alternative mechanisms for killing the intracellular mycobacteria (Figure 3B).

Given their importance in the development of immunity, we next wanted to examine the role of neutrophils during BCG vaccination (32). Neutrophils are the first cells at the site of infection and are responsible for initiating immune responses by activating other cells including macrophages and dendritic cells (33). As we hypothesized that the innate response responsible for BCG induced immunity significantly contributed to the killing of *M. tuberculosis*, we wanted to explore the role of neutrophils in the initial stimulation of the response, rather than the effector function of neutrophils that others have shown can function against mycobacteria (34). To examine the role of neutrophils in stimulating BCG induced immunity, mice were treated with anti-Ly6G antibody to deplete these cells prior to and during vaccination. The neutrophil population was allowed to return before infection on day 7, as antibody treatment was stopped on day 4 post-vaccination. Administering anti-Ly6G prior to and during BCG vaccination resulted in a 0.5 Log_10_ CFU reduction in mycobacteria compared to the 1 Log_10_ CFU reduction observed in the isotype control group (Figure 3C), and the same result was observed when neutrophils were depleted during vaccination and NK cells depleted during infection (Figure 3D). These data suggest that neutrophils played a significant role in establishing innate immunity, possibly through an early inflammatory response that initiates the reduction in mycobacterial burden. However, it does not imply any role for neutrophils in the direct killing of mycobacteria as the neutrophil populations were only depleted during vaccination and allowed to return during infection.

### Trained Innate Immunity Is Not a Factor in BCG Induced Killing of *M. tuberculosis* at 7 Days Post-vaccination

**Figure 4:**
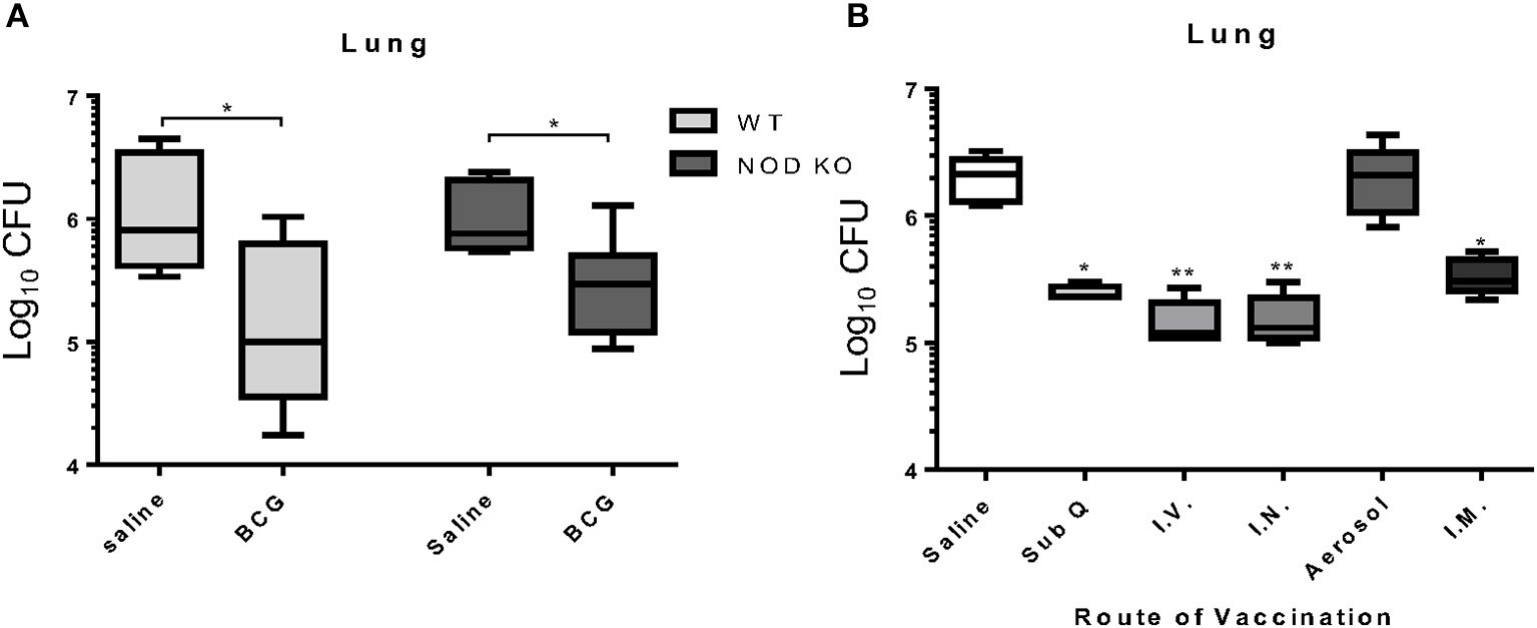
C57BL/6 mice deficient for both NOD1 and NOD2 (NOD KO) were vaccinated subcutaneously with either 5 × 10^4^ CFU BCG Pasteur or sterile pyrogen-free saline as controls. When vaccinated 7 days before infection, C57BL/6 NOD KO mice showed a capacity to significantly reduce the mycobacterial burden in the lungs **(A)**. In a head-to-head analysis, C57BL/6 mice were vaccinated with BCG by the routes; 5 × 10^4^ CFU subcutaneously (sub Q), 1 × 10^6^ CFU intravenous (I.V.), 1 × 10^7^ CFU intranasal (I.N.), 50–100 CFU low dose aerosol (Aerosol), and 5 × 10^4^ CFU intramuscular (I.M.), 7 days before infection. Mice are able to reduce the mycobacterial burden regardless of route of vaccination with the exception of low dose aerosol **(B)**. *N* = 5–6 mice per group, each performed once, *p ≤ 0.05. **p ≤ 0.01 (Kruskal Wallis test with Dunn *post-hoc* test).

In order to assess the importance of trained innate immunity to BCG induced protection against *M. tuberculosis* infection, we utilized a mouse model deficient in the NOD1 and NOD2 receptors. Even in the absence of the NOD receptors, BCG induced immunity resulted in a significant reduction in the mycobacterial burden in the lung similar to wild type mice (Figure 4A) indicating that NOD2-dependent trained innate immunity was not required for BCG induced protection against *M. tuberculosis* infection at day 7 post-vaccination. This suggested an alternative mechanism for induction of innate immunity and subsequent killing of mycobacteria. Kaufmann *et al*. recently released a report highlighting the importance of intravenous vaccine to induction of trained innate immunity (6). To determine if the route of vaccination affected BCG induced mycobacterial reduction within 7 days, mice were vaccinated in a head-to-head experiment, using five different routes; subcutaneous, intravenous, intranasal, low dose aerosol, and intramuscular. BCG vaccination induced a significant reduction in mycobacterial lung burden regardless of route of vaccination with the exception of aerosol vaccination (Figure 4B). These data suggest that BCG provided a potent immune response that was independent of the route. This was expected as aerosol vaccination deposited approximately 100 CFU BCG into the lungs of mice, which may have been insufficient to generate a protective immune response, or that the lung is prone to limit immune responses.

### BCG Replication Is Required for the Induction of Protective Immunity

**Figure 5:**
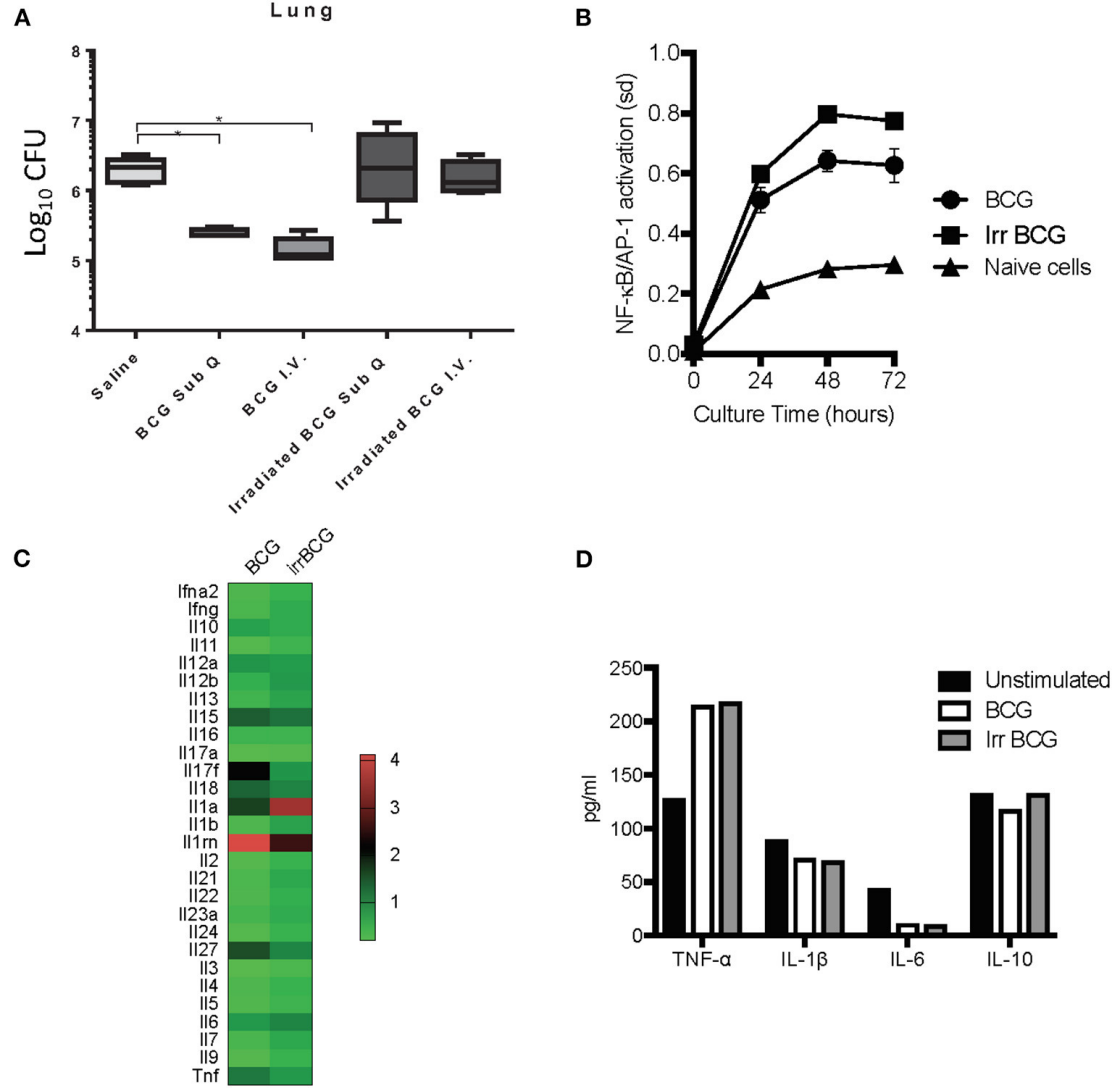
C57BL/6 mice were vaccinated with either live BCG or γ-irradiated BCG (irradiated BCG) subcutaneously or intravenously. γ-irradiated BCG was unable to stimulate a reduction in mycobacterial burden compared to live BCG which was able to reduce mycobacterial burden by 1 Log_10_ **(A)**. *N* = 5–6 mice per group, *p ≤ 0.05. The reporter RAW-Blue® cell line to assess NF-κB and AP-1 activation was used to assess the ability of either live BCG or γ-irradiated BCG to stimulate NF-kB and AP-1 signaling pathways. The activation of NF-kB and AP-1 signaling pathways was similar, regardless of whether the BCG was viable or non-replicating **(B)**. Cytokine mRNA expression was examined in C57BL/6-derived BMDM, stimulated with BCG for 24 h, using the RT2 profiler™ PCR array for mouse cytokines and chemokines **(C)**. Supernatants were also collected from these cultures and assessed for the production of protein cytokines TNF-α, IL-1β, IL-6, and IL-10 **(D)**. The *in vivo* data is a representative of multiple iterations with *N* = 5 mice per group. Data are representative of two *in vitro* studies.

Although trained innate immunity did not seem to be the principal component for BCG induced killing of *M. tuberculosis*, it did not preclude innate immune system involvement. BCG is an intracellular pathogen, and a known potent stimulator of the innate immunity (35) that has the ability to protect against non-mycobacterial diseases as well as certain cancers (11). Past studies have demonstrated that when given as a subcutaneous vaccine, live BCG can be found in various organs of mice including lungs (7) and bone marrow (6) There is also some suggestion that the numbers of BCG found are misrepresented as some live BCG may not replicate when taken out of the host (36). All of this suggests that BCG is able to survive in the endosomes of macrophages for extended periods of time, and some groups have reported this *in vitro* (37, 38), with evidence that BCG can inhibit phagosome maturation *in vivo* (39). However, it is unknown if induction of protective innate immunity depends on BCG remaining viable after inoculation and whether it needs to replicate. To address this question mice were vaccinated subcutaneously with either viable 5 × 10^4^ CFU BCG Pasteur or an equivalent dose of γ-irradiated BCG Pasteur. In addition, another cohort of mice were inoculated with either viable BCG or γ-irradiated BCG intravenously to determine if alternate routes worked as effectively as subcutaneous vaccination. Our findings indicated that the ability of BCG to actively replicate played a role in reducing the mycobacterial burden as γ-irradiated BCG inoculated groups did not produce the same level of mycobacterial killing, regardless of the route (Figure 5A). If BCG replication was important for the induction of strong innate immunity, it will be important to identify the mechanisms and how this translates to humans as BCG vaccines used in humans are lyophilized and coupled with improper storage can lead to only around 30% viability (40).

Given that γ-irradiated BCG did not induce protective immunity at day 7 post-vaccination, we wanted to determine if this was due to the inability of γ-irradiated BCG to activate antigen presenting cells such as macrophages. Using the reporter RAW-Blue® cell line to assess NF-κB and AP-1 activation, cells were cultured for 24, 48, and 72 h in the presence of either BCG or γ-irradiated BCG. NF-κB and AP-1 activation was similar between the two cultures, suggesting that γ-irradiated BCG was a good as viable, replicating BCG at activating macrophages (Figure 5B). To confirm our finding, we analyzed cytokine mRNA expression in C57BL/6-derived BMDM cultured with either BCG or γ-irradiated BCG, using the Qiagen RT2 Profiler™ PCR Array for Mouse Cytokines and Chemokines, and found no significant difference between the two stimuli in their ability to modulate mRNA expression of key pro- and anti-inflammatory cytokines (Figure 5C). Furthermore, at the protein level, production of TNF-α, IL-1β, IL-6, and IL-10 were similar between the two culture conditions (Figure 5D), reinforcing the fact that non-replicating BCG was a good as replicating BCG in producing key cytokines. Interestingly, the array data highlighted differences in mRNA expression for IL-17F, IL-27, IL-1a, and IL-1rn. Analysis of the ability of BCG and γ-irradiated BCG to induce phosphorylation of IRF3 and IRF7 signaling pathways by Western Blotting showed no significant difference in the activation of either pathway between viable and γ-irradiated BCG (data not shown).

### BCG Provides Protection in a LyzMcre Monocyte/Macrophage Knockout Mouse

**Figure 6:**
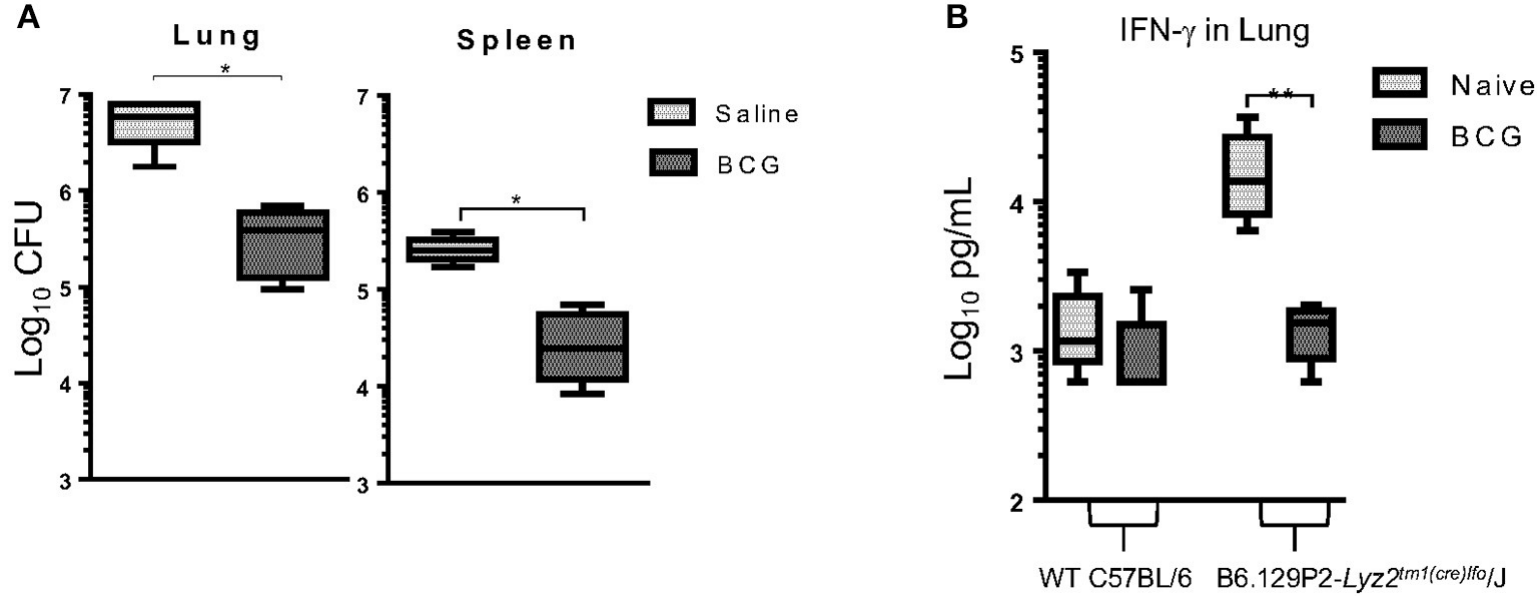
C57BL/6 LyzMcre-KO mice, unable to differentiate myeloid cells into mature granulocytes and monocytes, retained the ability to reduce mycobacterial burden when vaccinated with BCG, 7 days before infection **(A)**. Concentration of IFN-γ in lung supernatants from 30 days after infection as determined by ELISA **(B)**. *N* = 5 mice per group performed once and was then confirmed with a lung macrophage depletion experiment (Supplementary Figure 2). *p ≤ 0.05 **p ≤ 0.05 (*T*-test).

To further understand the role of monocytes/macrophages in BCG vaccination we utilized a LyzMcre knockout mouse in which the M-Lysozyme gene function has been abolished (41). The M lysozyme gene is responsible for the production of M lysozyme by macrophage cells which can be used to identify macrophages in addition to its highly antimicrobial properties (42). It is highly expressed during monocyte and macrophage development suggesting it plays a role in the process (43). Mice deficient of the M lysozyme gene function have been extensively characterized (44) and these data lend support to the notion that M lysozyme plays a role in differentiation and maturation (43, 45). These mice were vaccinated 7 days prior to infection and CFU and IFN-γ determined 30 days later. Again, BCG induced immunity resulted in a significant reduction in the mycobacterial burden in the lungs and spleen by 1 Log_10_ CFU (Figure 6A). These findings confirmed our prior observations in which Liposomal Clodronate (Encapsula NanoSciences LLC) was used to deplete macrophages (Supplementary Figure 2). This also validated previous findings by others that M lysozyme was not required to kill *M. tuberculosis*, despite lysozyme being a highly abundant antimicrobial peptide in pulmonary airways (46) as mycobacterial lipoproteins act as a lysozyme inhibitor (47). IFN-γ concentrations were elevated in the lungs of knockout mice 30 days after infection when compared to wild type mice. However, we observed that when vaccinated with BCG and mycobacterial killing was induced, the concentration of IFN-γ decreased to levels of those found in wild type mice (Figure 6B).

## Discussion

Our studies have demonstrated that BCG induced mycobacterial killing within 7 days of vaccination, in the absence of conventional T cells and related cytokines, NK cells, and in mice lacking normal monocyte development, so the question must be asked; what was responsible for the early mycobacterial killing induced by BCG vaccination? We chose to examine differences between BCG-vaccinated and non-vaccinated infected mice at day 30. There is a potential for T cell related immunity to be expressed at this time. However, studies have identified that a T cell response in the lung is not fully developed until a later time period (48). Additionally, our data using BCG-vaccinated mice, 30 days prior to infection, a time point at which effector adaptive immunity is being expressed, showed that the earliest time at which a significant difference was observed was at day 21 post-infection. These findings support previously published data showing that BCG vaccinated mice that had received the vaccine 16 weeks prior to *M. tuberculosis* Erdman infection (49). A delay in expression of immunity may be due to the inherent nature of the lung, a mucosal tissue site with its own intrinsic microenvironment. As demonstrated by others the pulmonary site is often prone to the induction of Th2-dominated responses (50, 51) and the host immune response may be inherently inhibitory in the lung after infection with *M. tuberculosis*, preventing BCG from being fully functional. Therefore, the best time to observe differences was beyond day 21 of infection. Similarly, previous studies showed delayed adaptive immune responses in the lung associated lymph nodes and subsequently the lung until ~3 weeks after infection (48). Our experiments showed an absence of effector T cell cytokines at this timepoint, highlighting the importance of other immune mechanisms to this observed mycobacterial reduction. It is also possible that *M. tuberculosis*-induced immune responses may be increasing with the progression of time that may add to the response or possibly compromise BCG-induced adaptive immunity, since BCG was given 1 week prior to infection. These studies highlight the fact that there is no defined point at which innate immunity ends and adaptive immunity begins, and that an overlap of responses is occurring throughout the vaccination and infection immune response.

We also hypothesized that natural killer (NK) cells may provide the early production of IFNγ required for killing M. tuberculosis as studies in the bovine model have previously demonstrated this (52–54). In humans, *in vitro* analyses suggest that BCG stimulated NKp44^+^CD56^bright^ and CD56^dim^ NK cells may have diverse modes of action, but no direct role for NK cells during infection (55–57). These studies, regardless of the host, demonstrated that BCG induced NK cell activation. Thus, in the current studies, we wanted to interrogate NK cells as a potential source of IFN-γ that could be stimulating macrophages to kill mycobacterium. To this end, NK cells were depleted during the infection phase to make sure they were not contributing to the mycobacterial killing. At this stage of the study we were focusing on the mechanism behind the actual mycobacterial killing, not development of immunity although both are important aspects.

It is unsurprising that neutrophils play an important role, as neutrophils are the second most abundant cell, only to lymphocytes, in broncho-alveolar lavage of human tuberculosis patients suggesting that there are strong chemo-attractants that cause them to extravasate into the lung during infection (58). Our observation of increased recruitment of neutrophils to the lungs after BCG vaccination only adds to the proposed roles for neutrophils in immunity to *M. tuberculosis* (59, 60). Other potential innate immune cells include mucosal-associated invariant T (MAIT) and innate lymphoid cells (ILCs). MAIT cells behave very similarly to T cells, and play a role in controlling pulmonary mycobacterial growth and other bacterial pathogens (61, 62). In addition, *M. tuberculosis* infection induces migration of MAIT cells into the lungs, suggesting a role for either bactericidal activity or in cell recruitment (63). Some data suggest that MAIT cells contribute to IFN-γ-mediated clearance of pulmonary *M. tuberculosis* and that blockade of PD-1 may enhance this ability, suggesting the potential for PD-1 based therapy (64), but it is unknown how this can influence innate immunity. Other ILCs are recruited to the lung by subcutaneous BCG vaccination (65), which supports our findings that indicated the presence of an immune response in the lungs 7 days after BCG vaccination. There has also been some suggestion that type 3 ILCs parallel the function and cytokine production of CD8^+^ T cells, and may rapidly respond to BCG in the lungs (66). Identifying how ILCs function in relation to BCG vaccination and induce mycobacterial killing will be important for vaccine development, as these cells may play a critical role in establishing vaccine-mediated pulmonary immunity.

To better understand the importance of BCG viability, we focused our attention on the effect of γ-irradiated BCG on macrophages. Our data demonstrated for the first time that BCG was not able to induce a 1 Log_10_ CFU reduction. We asked if it was due to the inability of γ-irradiated BCG to trigger vital signaling pathways required to activate macrophages, but our data suggested that γ-irradiated BCG was as good as replicating BCG to activate key cytokine activation pathways. Comparative targeted mRNA analysis of gene expression between BCG and γ-irradiated BCG stimulated BMDM showed differences in the IL-17F, IL-27, IL-1a, and IL-1rn, genes, some of which have been shown by others to be up-regulated in BCG stimulated human peripheral blood mononuclear cell cultures (67). IL-17F, produced by Th17 helper T cells, type 3 innate immune cells (ILCs), γδT cells, NK T cells, and CD8^+^ T cells, and by activated monocytes is a pro-inflammatory cytokine involved in host defense against bacterial and fungal infection (68, 69). The current set of studies identified IL-17F as a possible requirement for *M. tuberculosis* killing in the absence of a T cell response, but further data is lacking. IL-1β and IL-1RN were identified as major pro-inflammatory cytokines induced by BCG (70). Other studies have highlighted the importance of bacterial mRNA to initiation of the immune response suggesting that live bacteria stimulate a more potent immune response than dead (71, 72). As our analysis found no critical difference in cytokine production in cells after stimulation with live and γ-irradiated BCG, we concluded that the immunity initiating capacity was equivalent between the two.

Of particular note was our finding that BCG vaccination in LyzMcre KO mice caused a significant reduction in CFU. These mice have only a fully intact alveolar macrophage population and partially intact peripheral blood monocytes and neutrophils (44). It was also interesting that unvaccinated LyzMcre KO mice had increased IFN-γ production together with increased CFU. This further supports the fact that IFN-γ is not required for mycobacterial killing, as alveolar macrophages and IFN-γ would have been present. Altogether, our data support a model in which neutrophils function with monocytes (Figure 2) and alveolar macrophages to induce killing of mycobacterium. Additionally, serine proteases could function in place of IFNg which have been observed to induce killing of mycobacteria when produced by neutrophils (73) or monocytes (74, 75).

Finally, our results indicated that NOD2 dependent trained innate immunity was not a requirement for a reduction in mycobacterial lung burden. Recent reports have suggested that trained innate immunity was induced optimally through intravenous vaccination. Thirty days prior to infection (3, 6). As our study involving different routes of vaccination demonstrated, the induction of mycobacterial killing was not dependent on route and occurred within 7 days supporting the fact that our observations do not fit the paradigm of trained innate immunity and may suggest a mechanism prior to induction of trained immunity. A recent study in the non-human primate (NHP) indicated that I.V. BCG was markedly superior than other vaccination routes in protecting NHPs against infection (76). although this is likely the persistence of adaptive immunity. Our data suggest the potential for an unconventional innate immune mechanism for killing *M. tuberculosis* that is induced by BCG early, or within 7 days of vaccination. Our future studies plan to elucidate these mechanisms and hope to learn new ways to induce killing of *M. tuberculosis* through vaccination.

The development of a functional vaccine for tuberculosis is critical to reducing the spread and numbers of people who suffer from the disease. Many vaccines currently in trials focus on the stimulation of a strong T cell response (77), However, the data presented here provides evidence to support further development of other types of immune responses with a vaccine that may effectively reduce mycobacterial burden and, potentially, the mortality associated with active tuberculosis infection. The evidence here suggests a mechanism behind the success of the BCG vaccine in preventing disseminated infection in children. Perhaps the future of the BCG vaccine relies in this type of systemic immune response.

## Data Availability Statement

The datasets generated for this study can be found in the GEO GSE139864.

## Ethics Statement

The animal study was reviewed and approved by Colorado State University, Institutional Animal Care and Use Committee.

## Author Contributions

TB planned, performed the majority of the experiments, and wrote the manuscript. AAI planned the experiments and assisted in writing the manuscript. JM, EC, LI, CH, AJI, and FS performed various aspects of the experiments. All authors contributed to the article and approved the submitted version.

## Conflict of Interest

The authors declare that the research was conducted in the absence of any commercial or financial relationships that could be construed as a potential conflict of interest.
