# Supplementary material for: Characterizing the BCG Induced Macrophage and Neutrophil Mechanisms for Defense Against Mycobacterium tuberculosis
Source: Front Immunol. 2020 Jun 18;11:1202. doi: 10.3389/fimmu.2020.01202 (PMC7314953; doi:10.3389/fimmu.2020.01202)
Supplement: Supplementary file 1 [file Data_Sheet_1.pdf]

Supplementary Table 1

Marker	Fluorophore	Isotype	Clone	Vendor
CD11b	Alexa Fluor 700	Rat IgG2b	M1/70	BD Biosciences
CD11c	BV510	Arm Ham IgG1	HL3	BD Biosciences
Siglec F	PercP cy5.5	Rat IgG2a	E50-2440	BD Biosciences
Ly6G	PEcy7	Rat IgG2b	RB6-8C5	Tonbo Biosciences
Ly6C	APC	Rat IgG2a	1G7.G10	Miltenyi Biotec
F4/80	PE	Rat IgG2b kappa	BM8.1	Tonbo Biosciences
I-A[b] MHCII	BV421	Mouse IgG2a	AF6-120.1	BD Biosciences
CD14	FITC	Mouse IgG1 kappa	X54-5/7.1	Biolegend
NK 1.1	PE	Mouse IgG2a kappa	PK136	Tonbo Biosciences

Supplementary Table 1

Details of antibodies used for flow cytometry in the current studies.

Supplementary Figure 1

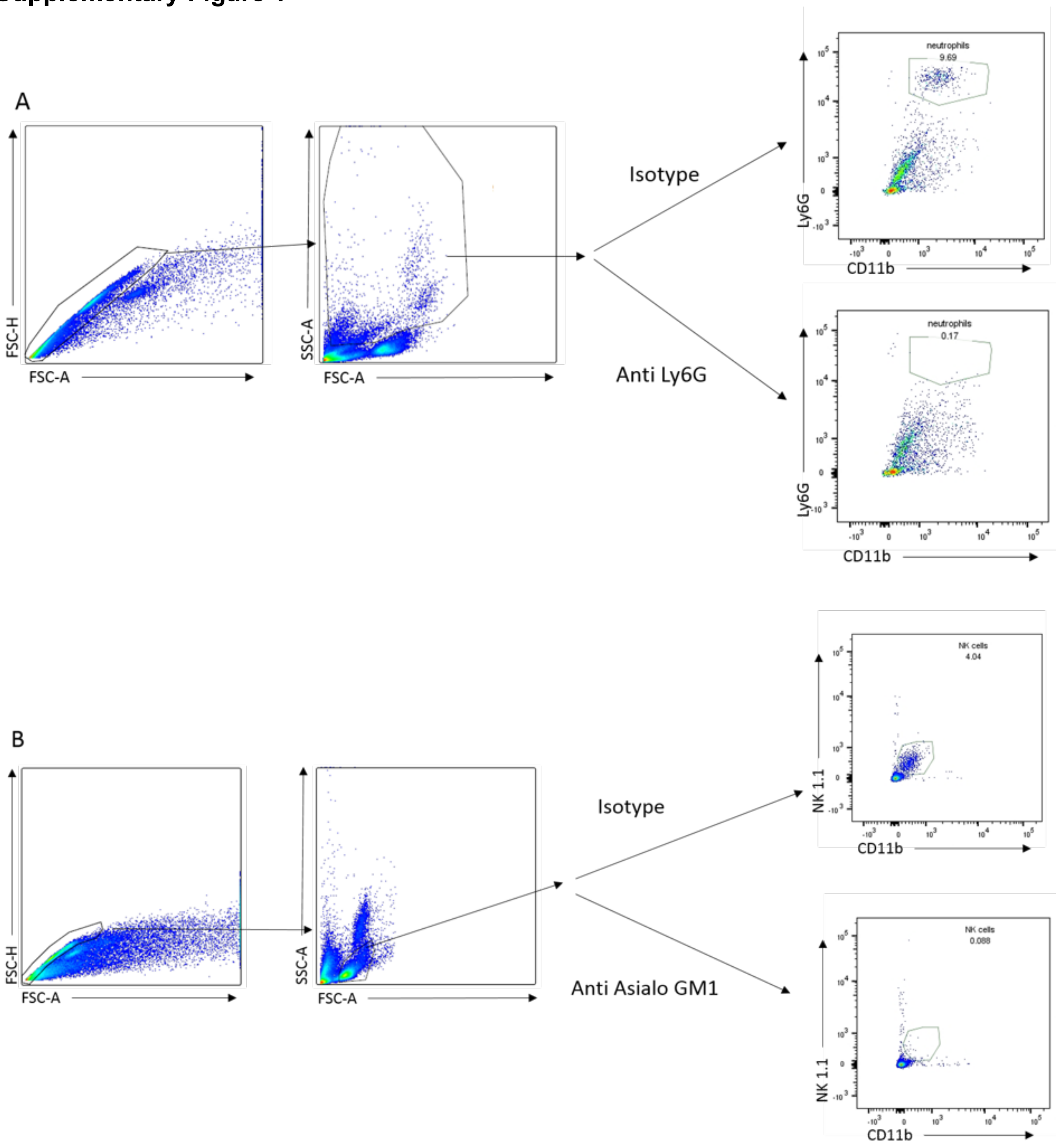

Supplementary Figure 1

Flow cytometric confirmation of neutrophil depletion using a Ly6G antibody (**A**) and of natural killer cell depletion using an NK1.1 antibody (**B**). Peripheral blood mononuclear cells were used to examine the efficacy of depletion.

Supplementary Figure 2

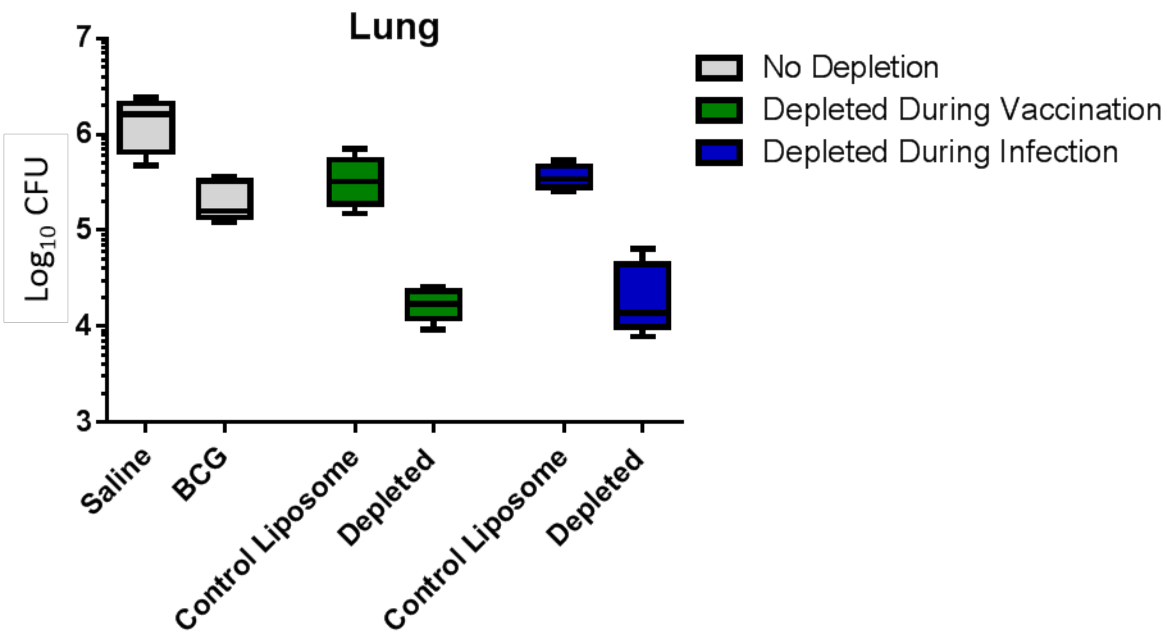

Supplementary Figure 2

C57BL/6 depleted of monocytes/macrophages through intravenous liposomal clodronate depletion still are protected by subcutaneous BCG vaccination 7 days prior to low dose aerosol infection. Depletions were carried out during BCG vaccination and during H37Rv infection.

Supplementary Figure 3

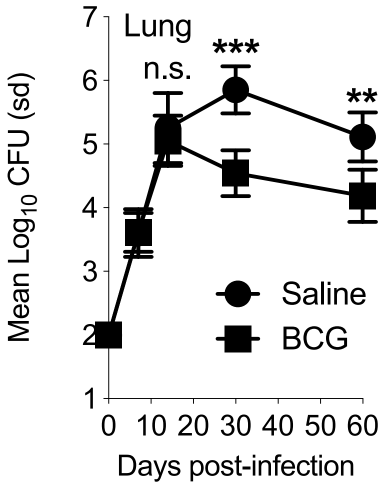

Supplementary Figure 3

C57BL/6 mice were vaccinated subcutaneously with $5x10^4$ CFU BCG, rested for 30 days and then infected with a low dose aerosol of *M. tuberculosis* H37Rv. The Log_{10} CFU was determined at days 0, 7, 14, 30 and 60 post infection. Data are representative of two separate experiments. Experiments were performed with N=4-5 mice per group per time point. **p≤0.01, ***p≤0.001, n.s. = not significant, using the Student *t*-test.
